# Experimental validation and pan-cancer analysis identified COL10A1 as a novel oncogene and potential therapeutic target in prostate cancer

**DOI:** 10.18632/aging.205337

**Published:** 2023-12-21

**Authors:** Shengxian Xu, Dongze Liu, Zheng Qin, Zhengxin Liang, Hongbo Xie, Bocun Yi, Kaibin Wang, Gaoteng Lin, Ranlu Liu, Kuo Yang, Yong Xu, Hongtuan Zhang

**Affiliations:** 1 Department of Urology, National Key Specialty of Urology, Second Hospital of Tianjin Medical University, Tianjin 300211, China; 2 Department of Oncology, Second Hospital of Tianjin Medical University, Tianjin 300211, China

**Keywords:** COL10A1, pan-cancer, prognosis, immune analysis, prostate cancer

## Abstract

Background: Type X collagen (COL10) is a homologous trimeric non-fibrillar collagen found in the extracellular matrix of human tissues, and it exhibits a distinctive white appearance. Type X collagen α1 chain (COL10A1) is a specific cleaved fragment of type X collagen. However, the expression, prognostic significance, clinicopathological attributes and immune-related associations of COL10A1 in prostate cancer as well as in pan-cancer contexts remain poorly understood.

Methods: Using bioinformatic analysis of data from the most recent databases (TCGA, GTEx and GEO databases), we have extensively elucidated the role played by COL10A1 in terms of its expression patterns, prognostic implications, and immune efficacy across a pan-cancer spectrum. Subsequently, the biological functions of COL10A1 in prostate cancer were elucidated by experimental validation.

Results: Our findings have confirmed that COL10A1 was highly expressed in most cancers and was associated with poorer prognosis in cancer patients. Immune correlation analysis of COL10A1 in various cancers showed its significant correlation with Tumor mutational burden (TMB), microsatellite instability (MSI) and immune cell infiltration. In addition, knockdown of COL10A1 in prostate cancer resulted in a substantial reduction in the proliferation, migration, and invasive potential of prostate cancer cells.

Conclusion: Our pan-cancer analysis of COL10A1 gene provided novel insights into its pivotal role in cancer initiation, progression, and therapeutic implications, underscoring its potential significance in prognosis and immunotherapeutic interventions for cancer, particularly prostate cancer.

## INTRODUCTION

Collagen type X (COL10) belongs to the collagen family. The COL10A1 gene encodes the alpha chain of collagen type X. Collagen type X is a short-chain collagen expressed by mast chondrocytes during endochondral ossification [1]. Collagen type X is synthesised and secreted by mast chondrocytes at the site of endochondral ossification, and it is generally present in the body as a liquid [2]. Therefore, it is generally present in the human body at sites of active cartilage ossification such as the sternum, craniofacial cartilage and long bones. In the normal state, the level of type X collagen decreases with the cessation of skeletal development [3, 4]. Therefore, abnormally high level of COL10A1 may predict the development of malignant tumors. In recent years, many researches have pointed out that in some solid malignant tumors such as breast cancer, colorectal cancer, gastric cancer, and lung adenocarcinoma, elevated levels of type X collagen expression were closely associated with tumor growth, proliferation, migration, and poor prognosis [5–8]. However, the role of this gene in pan-cancer has not been elucidated.

In recent years, the tumor microenvironment (TME) has garnered substantial attention among the scientific community [9]. The TME encompasses a complex interplay of tumor cells, immune cells, and stromal cells, providing a conductive milieu for the tumor development and survival. TME has emerged as an important factor in cancer development and progression [10]. Infiltrating immune cells (e.g., tumor-associated regulatory T cells, B cells, macrophages and natural killer (NK) cells) in the TME play an important role in tumor development and progression and exhibit a high degree of heterogeneity and plasticity. T cells and NK cells combat tumor growth through different mechanisms such as the releasing of perforin and granzymes to induce apoptosis, while M2 macrophages are immunosuppressive and can induce the immune escape of cancer cells [11, 12]. Recently, immunotherapy has emerged as a promising option to advance cancer treatment [13]. However, cancer immunotherapies have yet to meet the expectations for satisfactory outcomes. Therefore, it is of vital importance to explore new therapeutic targets for cancer immunotherapy to bridge the gap between the growing demand for cancer treatment and limited treatment options.

To our knowledge, our study was the first to address issues in investigating the prognostic and immunological significance of COL10A1 in a pan-cancer context. The expression of COL10A1 and its prognostic significance across different cancer types was assessed using various databases including TCGA, PrognoScan, Kaplan-Meier plotter, and TIMER. Then, we examined the correlation between COL10A1 expression levels and infiltration of immune cells, immune and stromal cell scores, TMB, MSI, and immunotherapy cohorts in pan-cancers. Finally, Gene Expression Omnibus (GEO) datasets were utilized to validate COL10A1 expression levels in Prostate adenocarcinoma (PRAD).

## RESULTS

### COL10A1 expression profile in human normal and tumor tissues

**Figure 1:**
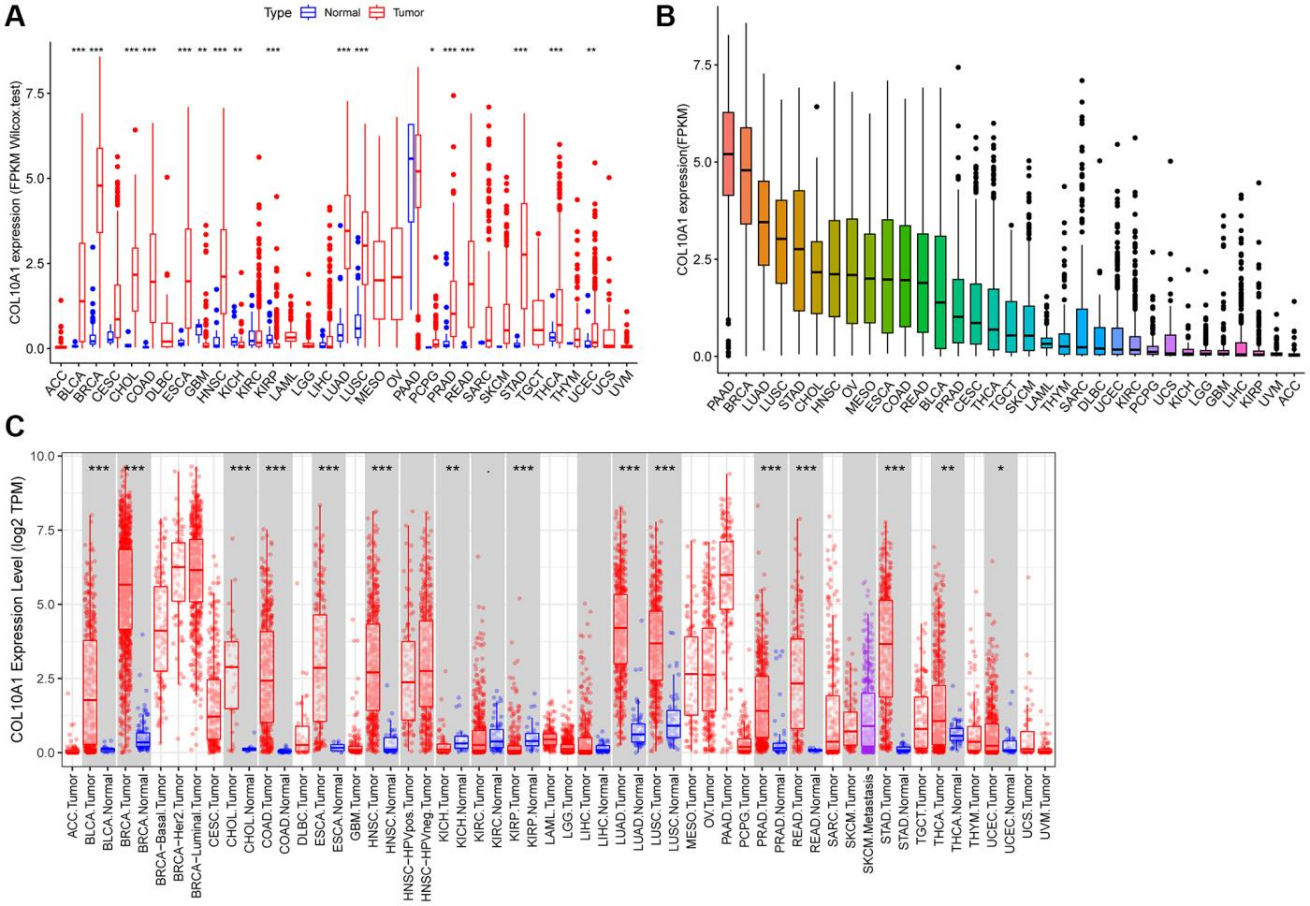
**COL10A1 expression in different cancers.** Differential expression analysis of COL10A1 between tumor and normal groups in 33 cancers (**A**). Mean expression of COL10A1 in 33 cancers (from high to low) (**B**). Pan-cancer expression profile of COL10A1 from the TIMER database (**C**).

COL10A1 expression data was acquired from 33 paired tumor and normal samples from TCGA database, and the expression disparities were assessed using TCGA and GTEx databases. The full nomenclature and abbreviations for the 33 cancers under investigation in this study can be found delineated in Supplementary Table 1. In the TCGA database, the transcriptome data suggested that the expression of COL10A1 was remarkably upregulated in Bladder urothelial carcinoma (BLCA), Breast invasive carcinoma (BRCA), Cholangiocarcinoma (CHOL), Colon adenocarcinoma (COAD), Esophageal carcinoma (ESCA), Head and neck squamous cell carcinoma (HNSC), Lung adenocarcinoma (LUAD), Lung squamous cell carcinoma (LUSC), Pheochromocytoma and Paraganglioma (PCPG), Prostate adenocarcinoma (PRAD), Rectum adenocarcinoma (READ), Stomach adenocarcinoma (STAD), Thyroid carcinoma (THCA) and Uterine corpus endometrial carcinoma (UCEC), whereas its expression was low in Glioblastoma multiforme (GBM), Kidney chromophobe (KICH) and Kidney renal papillary cell carcinoma (KIRP) (Figure 1A). Figure 1B showed the average expression level of COL10A1 in 33 different cancer types (from the highest to the lowest).

Interestingly, twenty-eight out of 34 cancer types showed significant differences in COL10A1 expression when data from the TCGA and GTEx databases were compared. Adrenocortical carcinoma (ACC), Glioblastoma multiforme (GBM), Glioma (GBMLGG), Renal clear cell carcinoma (KIRC), Brain Lower Grade Glioma (LGG) and Uterine Carcinosarcoma (UCS) were excluded. COL10A1 remained at high levels in certain tumor types such as ALL, BRCA, BLCA, CESC, COAD, READ, CHOL, ESCA, HNSC, LAML, LUAD, LUSC, LIHC, OV, PAAD, PCPG, PRAD, READ, SKCM, STAD, THCA, TGCT and UCEC, while it was significantly downregulated in KICH and KIRP (Supplementary Figure 1).

Lastly, the expression disparities of COL10A1 in various cancer types were assessed using TIMER database (Figure 1C). Consistent with previous analyses using TCGA and GTEx, the expression of COL10A1 was prominently higher in most tumor samples including BLCA, BRCA, CHOL, COAD, ESCA, HNSC, LUAD, LUSC, PRAD, READ, STAD, THCA and UCEC compared to normal tissues, while it was significantly downregulated in KICH and KIRP (Figure 1C).

### Correlation between the expression of COL10A1 and the prognosis of patients

**Figure 2:**
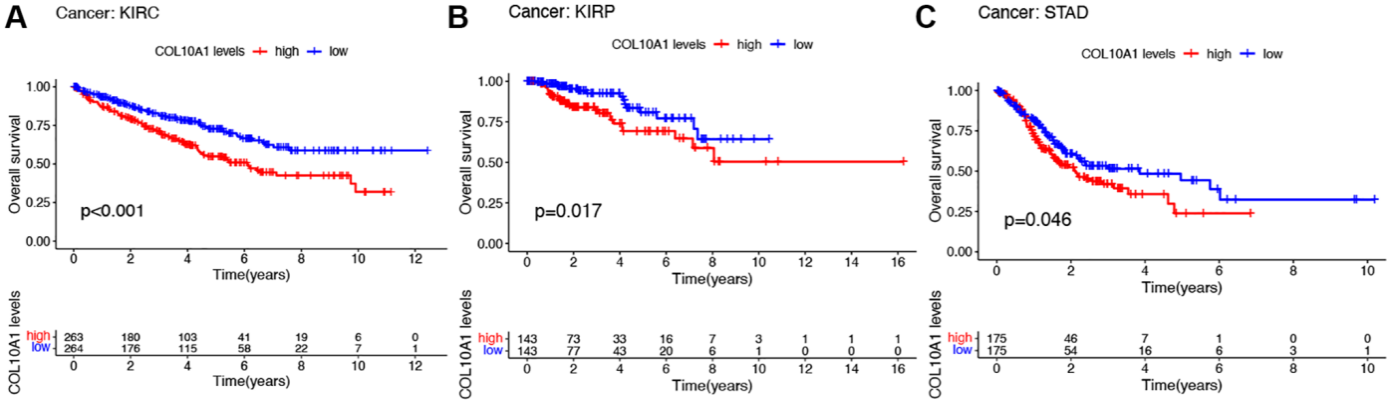
**Kaplan–Meier survival analyses of the prognostic value of COL10A1 expression level for OS in different cancer types.** OS according to high and low COL10A1 expression in KIRC, KIRP and STAD from TCGA database (**A**–**C**).

To better understand the prognostic significance of COL10A1 in cancer, the association between COL10A1 levels and multiple survival outcomes for each type of cancer, including OS, DSS, DFS and PFS was investigated. In KIRC, KIRP and STAD, a high level of COL10A1 expression was found to be associated with a shorter OS (Figure 2A–2C). Cox regression analysis indicated that elevated COL10A1 levels were associated with poorer OS in GBM (*p* = 0.046, HR = 1.343), KIRC (*p* = 1.60E-04, HR = 1.367), KIRP (*p* = 1.11E-11, HR = 4.262), PAAD (*p* = 0.004, HR = 1.197), STAD (*p* = 0.022, HR = 1.117), and UCEC (*p* = 0.039, HR = 1.267) (Supplementary Figure 2A).

**Figure 3:**
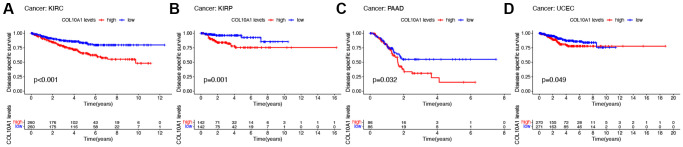
**Kaplan–Meier survival analyses of the prognostic value of COL10A1 expression level for DSS in different cancer types.** DSS according to high and low expression of COL10A1 in KIRC, KIRP, PAAD and UCEC from the TCGA database (**A**–**D**).

High COL10A1 expression correlated with shorter DSS in KIRC, KIRP, PAAD and UCEC in Kaplan-Meier survival curves (Figure 3A–3D). According to the cox regression outcomes, increased COL10A1 levels were correlated with shorter DSS in GBM (*p* = 0.017, HR = 1.423), KIRC (*p* = 1.27E-05, HR = 1.495), KIRP (*p* = 6.70E-12, HR = 4.986), PAAD (*p* = 0.004, HR = 1.226) and UCEC (*p* = 0.014, HR = 1.373) (Supplementary Figure 2B).

**Figure 4:**
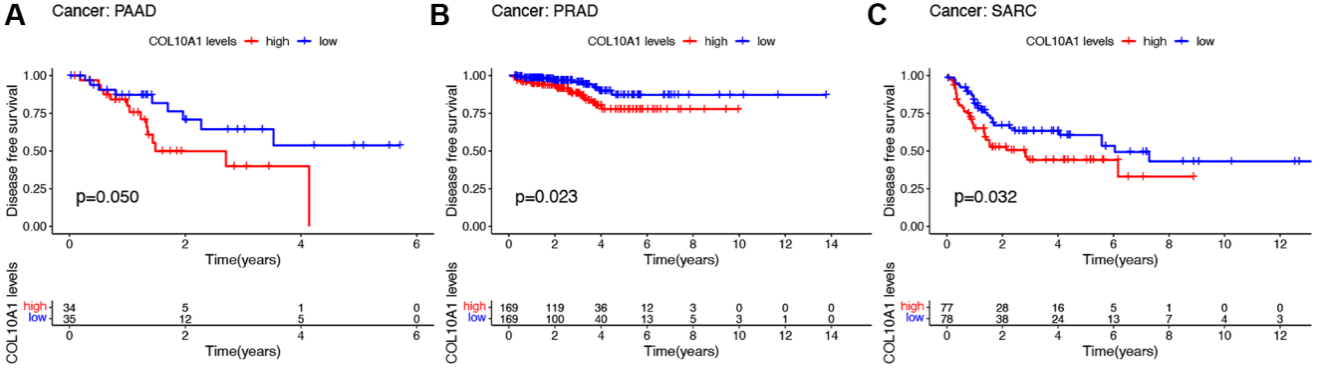
**Kaplan–Meier survival analyses of the prognostic value of COL10A1 expression level for DFS in different cancer types.** DFS according to high and low expression of COL10A1 in PAAD, PRAD and SARC from the TCGA database (**A**–**C**).

In terms of DFS, high level of COL10A1 expression correlated with poorer DFS in a large number of PAAD, PRAD, and SARC patients (Figure 4A–4C). Cox regression analysis showed that COL10A1 was a risk factor in CESC (*p* = 0.003, HR = 1.463), ESCA (*p* = 0.023, HR = 1.295), KIRP (*p* = 0.002, HR = 2.671), PAAD (*p* = 0.008, HR = 1.446) and STAD (*p* = 0.021, HR = 1.254), while it was a beneficial role in UCEC (*p* = 0.044, HR = 0.562) (Supplementary Figure 2C).

**Figure 5:**
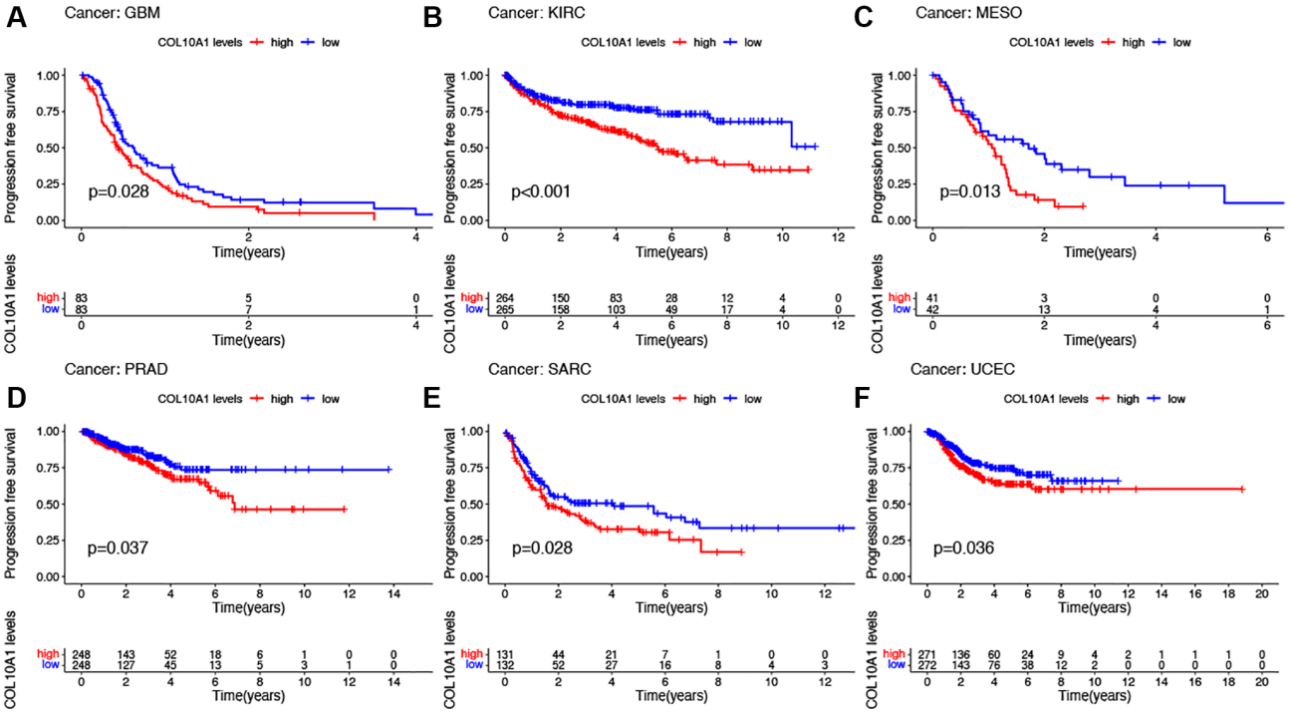
**Kaplan–Meier survival analyses of the prognostic value of COL10A1 expression level for PFS in different cancer types.** PFS according to high and low expression of COL10A1 in GBM, KIRC, MESO, PRAD, SARC and UCEC from the TCGA database (**A**–**F**).

Notably, in GBM, KIRC, MESO, PRAD, SARC and UCEC, the survival duration was markedly longer in the low expression level group than in the high expression level group (Figure 5A–5F). Cox regression showed that COL10A1 was associated with PFS in five cancer types: GBM (*p* = 1.43E-04, HR = 1.684), KIRC (*p* = 1.86E-05, HR = 1.409), KIRP (*p* = 7.48E-07, HR = 2.289), PAAD (*p* =0.006, HR = 1.172) and PRAD (*p* = 0.003, HR = 1.283) (Supplementary Figure 2D). To conclude, COL10A1 was a risk factor for all these cancer types mentioned above.

To observe the impact of COL10A1 on the prognosis of patients with different cancer types, Kaplan-Meier plotter was employed. It showed that COL10A1 overexpression was significantly associated with poorer OS in patients with BLCA, BRCA, KIRC, KIRP, LIHC, UCEC, SARC, while it remains the opposite for patients with THYM (Supplementary Figure 3).

Subsequently, PrognoScan was employed to evaluate the relationship between COL10A1 and the prognosis of each type of cancer (Supplementary Table 2). In particular, COL10A1 serves as a poor prognostic marker in bladder cancer (OS and DSS), brain cancer (OS), breast cancer (RFS and DFS), colorectal cancer (OS, DSS, and DFS), lung cancer (OS and RFS), ovarian cancer (OS and DFS) and soft tissue cancer (DFS). These data indicated that high COL10A1 expression were associated with poor clinical outcomes in cancer, especially in KIRC, KIRP, STAD, PAAD, SARC, PRAD and UCEC.

### Relationship between COL10A1 levels and clinicopathological features

**Figure 6:**
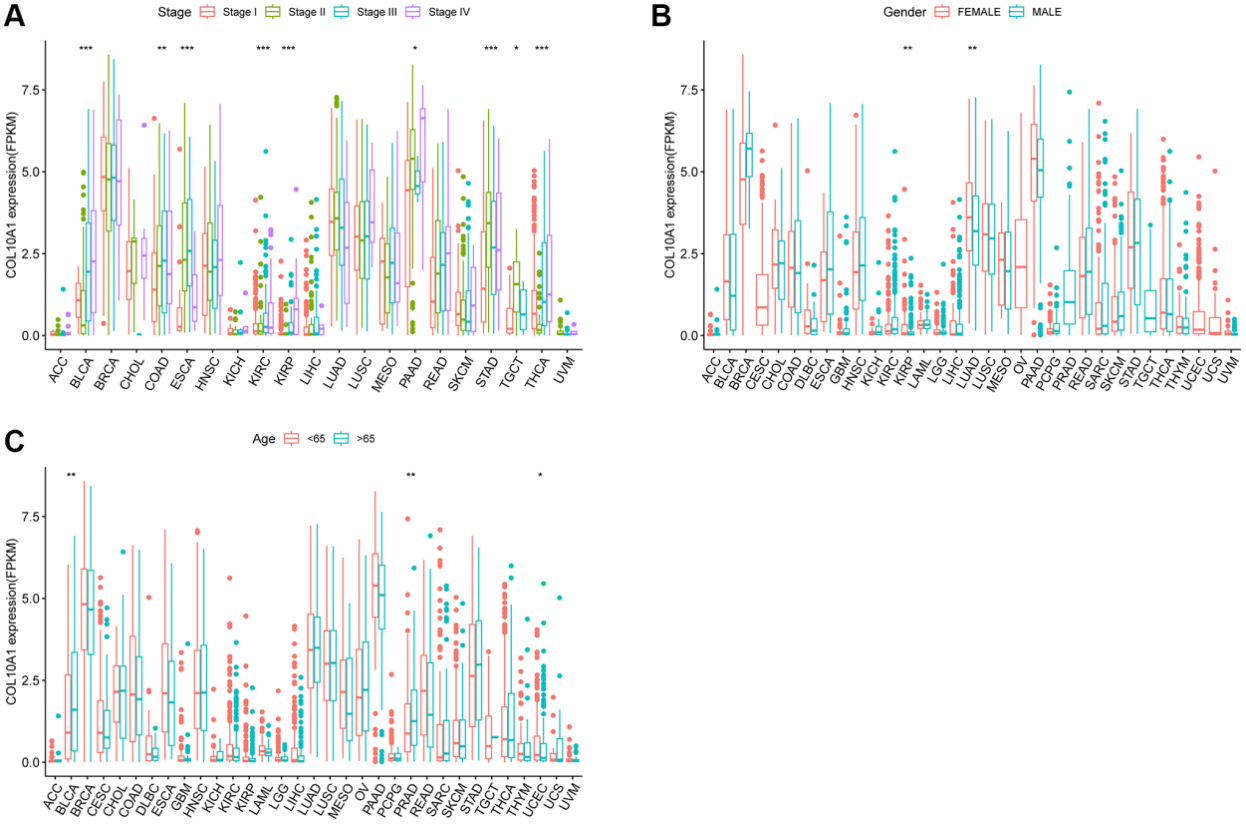
**Relationship between COL10A1 expression and clinical character.** Correlation between tumor stage and COL10A1 (**A**). Correlation between gender and COL10A1 (**B**). Correlation between age and COL10A1 (**C**). ^*^p < 0.05, ^**^p < 0.01 and ^***^p < 0.001.

High COL10A1 mRNA levels were correlated with more advanced tumor stage in BLCA (*p* < 0.001), COAD (*p* < 0.01), ESCA (*p* < 0.001), KIRC (*p* < 0.001), KIRP (*p* < 0.001), PAAD (*p* < 0.05), STAD (*p* < 0.001), TGCT (*p* < 0.05) and THCA (*p* < 0.001) (Figure 6A). Notably, a pronounced gender-related disparity in COL10A1 expression levels was observed in KIRP and LUAD. COL10A1 levels were higher in females than males in KIRP (*p* < 0.01) and LUAD (*p* < 0.01) (Figure 6B) group. In comparison to younger patients, COL10A1 was highly expressed in BLCA (*p* < 0.01), PRAD (*p* < 0.01) and low in UCEC (*p* < 0.05) (Figure 6C).

### Analysis of the mutation landscape of COL10A1 in various cancer types

**Figure 7:**
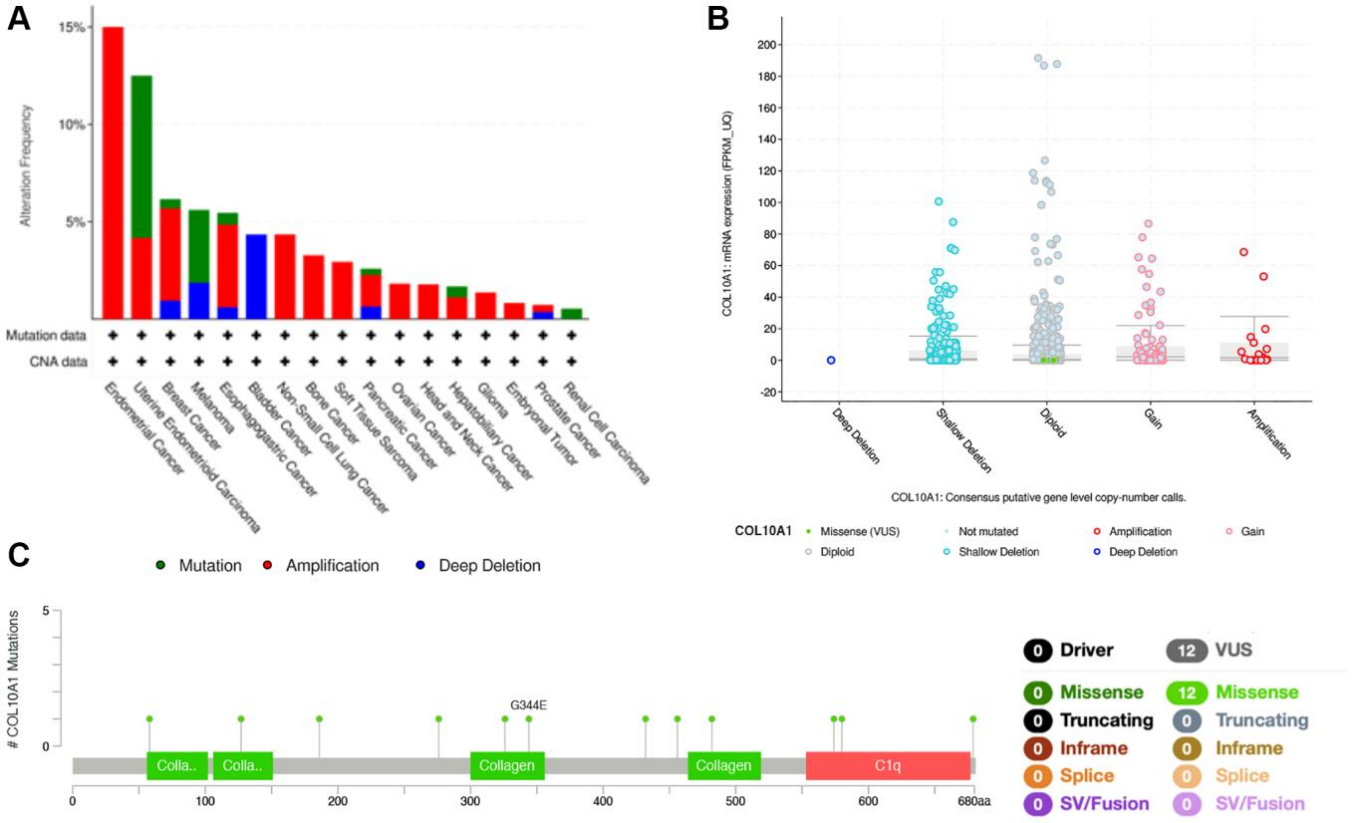
**COL10A1 mutation status according to the cBioPortal database.** COL10A1 mutation frequency in pan-cancer examined by the cBioPortal database (**A**). COL10A1 mutation level in pan-cancer examined by the cBioPortal database (**B**). Mutation diagram of COL10A1 in pan-cancer was examined by the cBioPortal database (**C**).

COL10A1 copy number alterations were assessed by cBioPortal database. Among various human cancers, Endometrial Cancer had a much higher mutation frequency (Figure 7A). Interestingly, in BLCA, deep deletion was the only type of genetic alteration. Moreover, it was found that amplification was the most common type of mutation at the pan-cancer level (Figure 7B). Meanwhile, using the cBioPortal database, COL10A1 mutations was screened in different cancers and 12 missense sites between amino acids 0 and 680 was identified (Figure 7C).

### Pan-cancer study of the relationship between COL10A1 and tumor immunity

**Figure 8:**
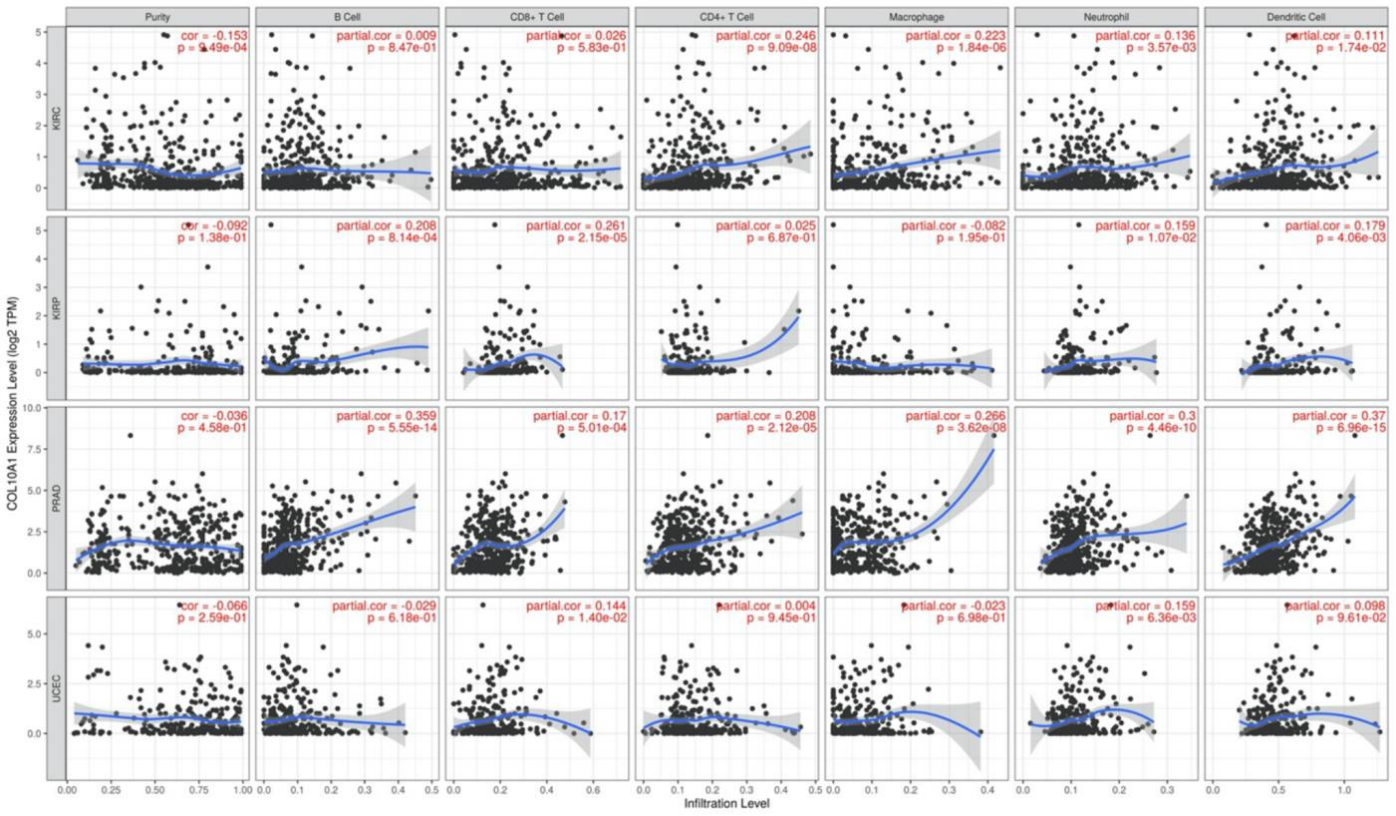
**Correlation of COL10A1 expression levels with immune cell infiltration in the TIMER database.** Correlation between six immune cell infiltration scores (B cell, CD4+ T cell, CD8+ T cell, neutrophil, macrophage, dendritic cell) and COL10A1 mRNA expression in Kidney renal clear cell carcinoma (KIRC), Kidney renal papillary cell carcinoma (KIRP), Prostate adenocarcinoma (PRAD), and Uterine corpus endometrial carcinoma (UCEC). (Spearman correlation test, *p* < 0.05 was considered significant).

**Figure 9:**
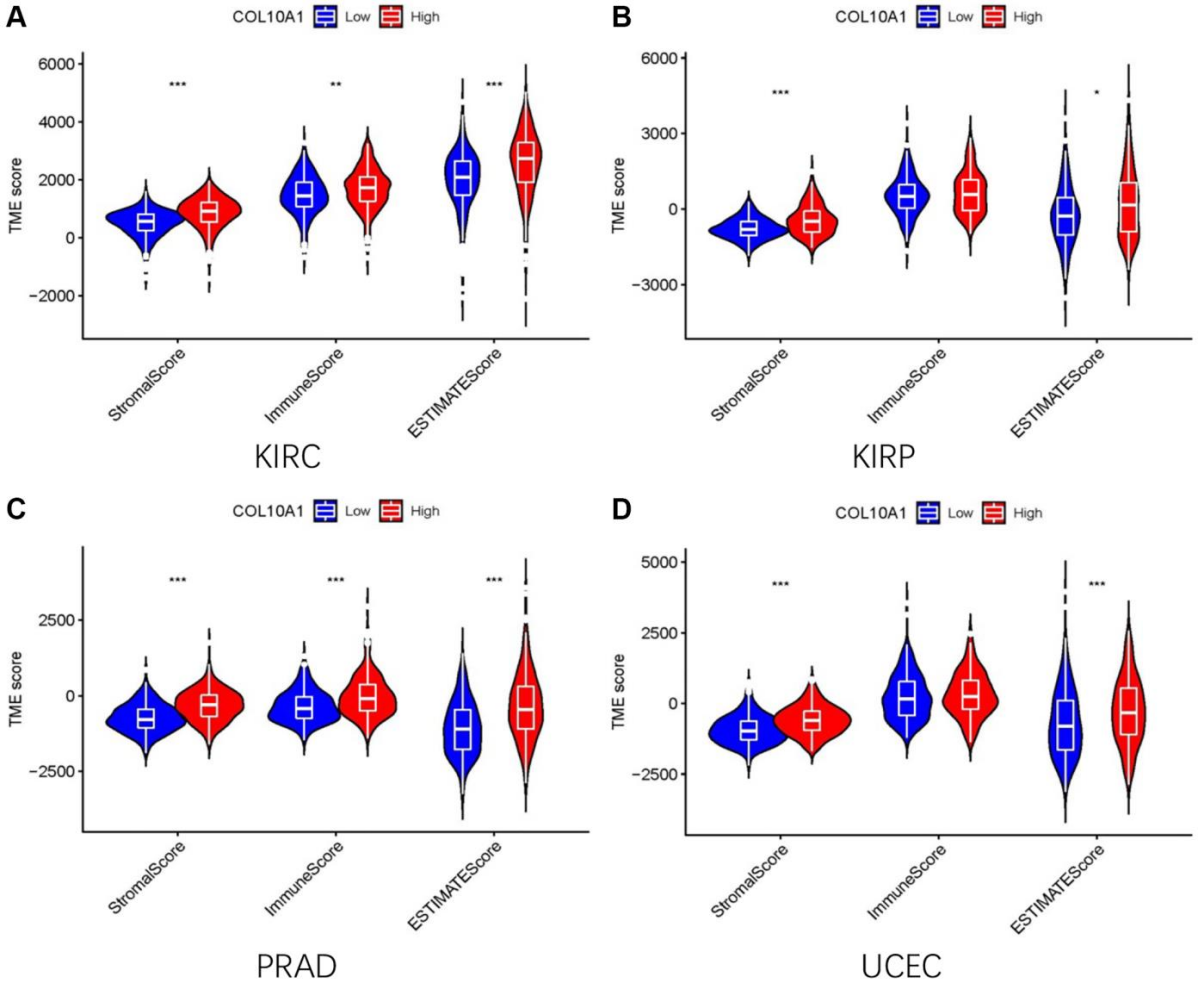
**The high and low expression groups of COL10A1 were correlated with the ESTIMATE score, which includes the stromal score (indicating the presence of stromal cells in the tumor tissue), immune score (representing the infiltration of immune cells in the tumor tissue), and the purity of the tumor.** Analysis of differences in stromal scores, immune scores and ESTIMATE scores between high and low COL10A1 expression groups in four cancers (KIRC, PRAD, KIRP, and UCEC) (**A**–**D**).

The relationship between immune cell infiltrated and COL10A1 levels in pan-cancer was assessed using the TIMER database (Figure 8). It is noteworthy that the level of all immune infiltrating cells in prostate cancer was positively correlated with COL10A1. The same correlation was also observed in BRCA. By contrast, there was no association between COL10A1 level and the infiltration of immune cells in UVM (Supplementary Figure 4). After a comprehensive analysis of gene expression, correlation of prognostic and clinical features, and immune cell infiltration, our team selected KIRC, KIRP, PRAD, and UCEC as the four most investigated four cancers for analysis. Higher COL10A1 expression was associated with higher stromal score, immune score and ESTIMATE score in KIRC and PRAD (Figure 9A, 9C), while only higher stromal score and ESTIMATE score were found associated with higher COL10A1 expression in KIRP and UCEC (Figure 9B, 9D).

The TISIDB database was then utilized to investigate the relationship between COL10A1 and immune and molecular features in various types of cancer. All of the tumor samples sourced from TISIDB database have been classified into 6 immunological subtypes, namely: C1, wound healing; C2, IFN-γ dominant; C3, inflammatory; C4, lymphocyte depleted; C5, immunologically quiet and C6, TGF-β dominant. Among these cancers, COL10A1 expression level was strongly associated with immune subtypes of BLCA, BRCA, CESC, COAD, HNSC, KICH, KIRC, KIRP, LIHC, LUAD, LUSC, MESO, OV, PAAD, PRAD, TGCT, THCA, UCEC and UVM (all *p* < 0.05; Supplementary Figure 5). COL10A1 expression was low in the C4 form in the majority of the 16 cancers. In KICH, COL10A1 was lowly expressed in C5, while it was expressed at low levels in type C3 in PRAD.

In addition, the expression of COL10A1 was markedly variable in the various molecular subtypes of the BRCA, COAD, ESCA, GBM, HNSC, KIRP, LGG, LIHC, LUSC, OV, PCPG, PRAD, STAD and UCEC (Supplementary Figure 6). These findings suggested a strong correlation between COL10A1 expression and both immune and molecular subtypes of diverse cancer types.

### The relationship between the expression of COL10A1 and TMB and MSI

**Figure 10:**
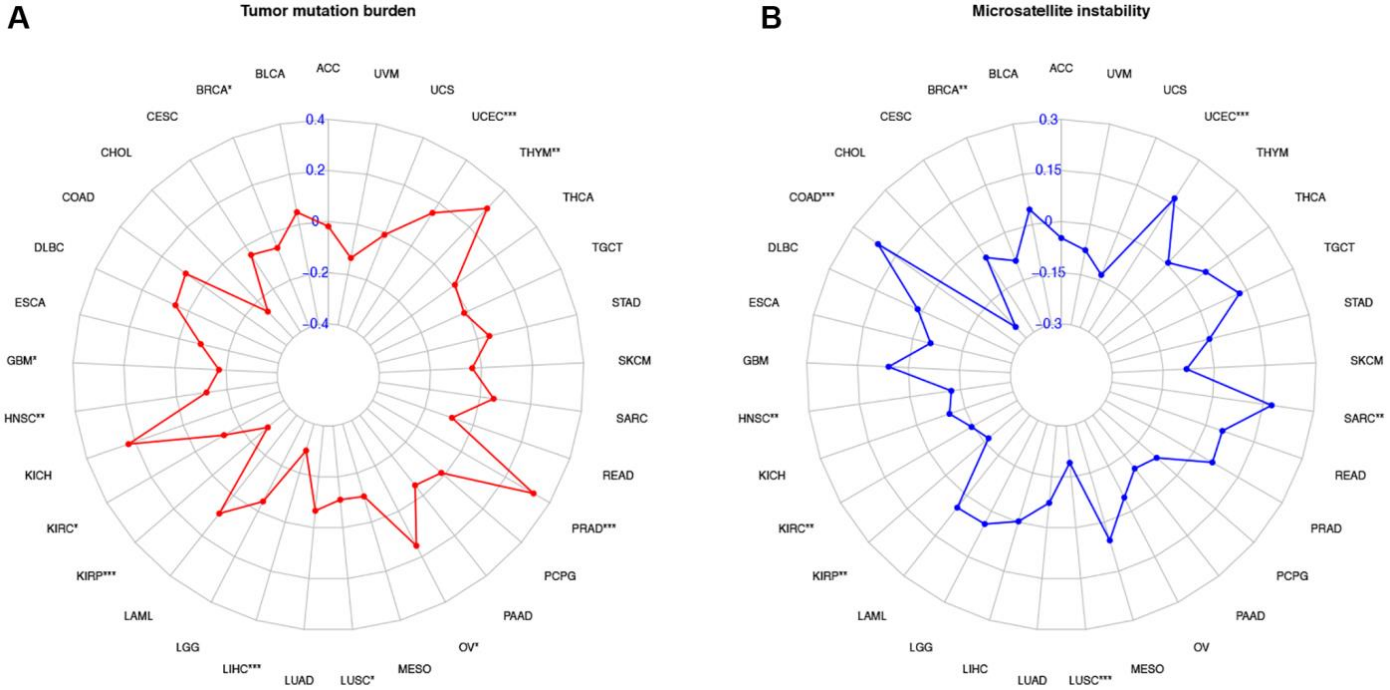
**Correlation of COL10A1 expression with tumor mutation burden (TMB) and microsatellite instability (MSI) in multiple cancers.** Correlation between TMB and COL10A1 expression (**A**). Correlation between MSI and COL10A1 expression (**B**). The Spearman correlation coefficients are shown above the bar graphs. (Spearman Correlation test, *p* < 0.05 was considered significant, ^*^p < 0.05, ^**^p < 0.01, ^***^p < 0.001).

The association between the expression of COL10A1, TMB and MSI was also investigated. The COL10A1 expression level in OV, PRAD, THYM and UCEC were positively associated with TMB, whereas a negative association was observed in BRCA, GBM, HNSC, KIRP, KIRC, LIHC and LUSC (*p* < 0.05; Figure 10A). In the analysis of MSI, COAD, SARC and UCEC were positively correlated in the group (*p* < 0.05; Figure 10B). BRCA, HNSC, KIRC, KIRP and LUSC were negatively correlated in the group (*p* < 0.05; Figure 10B).

### Immunotherapy

**Figure 11:**
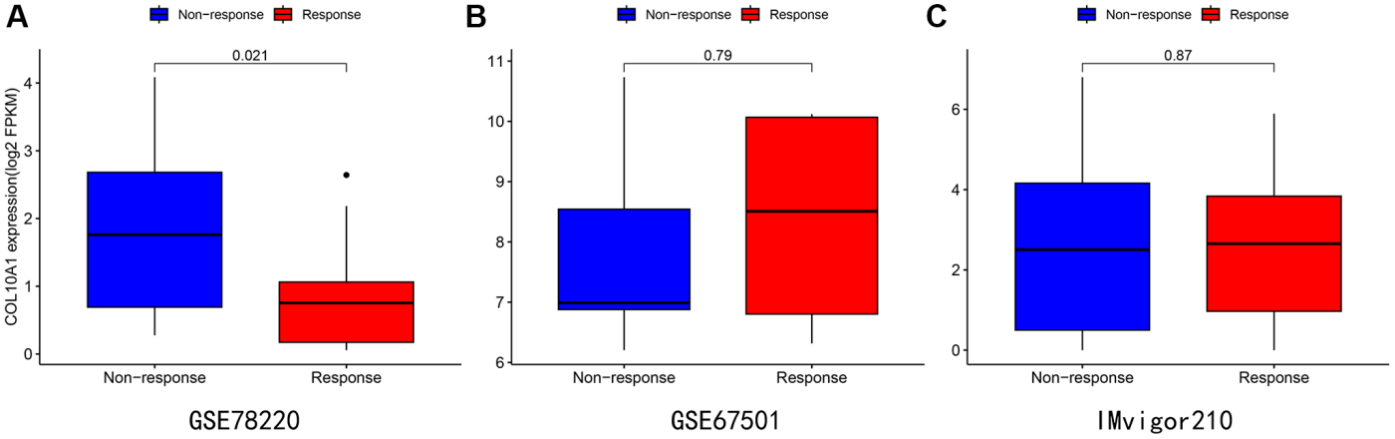
Immunotherapy analysis of melanoma (**A**), renal cancer (**B**), and urothelial epithelial tumor (**C**) in data from the GSE78220 (**A**), GSE67501 (**B**), and IMvigor210 database (**C**).

After analyzing three different treatment cohorts, it was found that people with low COL10A1 expression that people with low expression level of COL10A1 were more responsive to atezolizumab treatment in advanced melanoma carcinoma (GSE78220 cohort downloaded from previously published studies) (Figure 11A). Nevertheless, the differences were not statistically significant in cohorts GSE67501 (*p* = 0.79) and IMvigor210 (*p* = 0.87) (Figure 11B, 11C).

### Further validation in prostate adenocarcinoma

**Figure 12:**
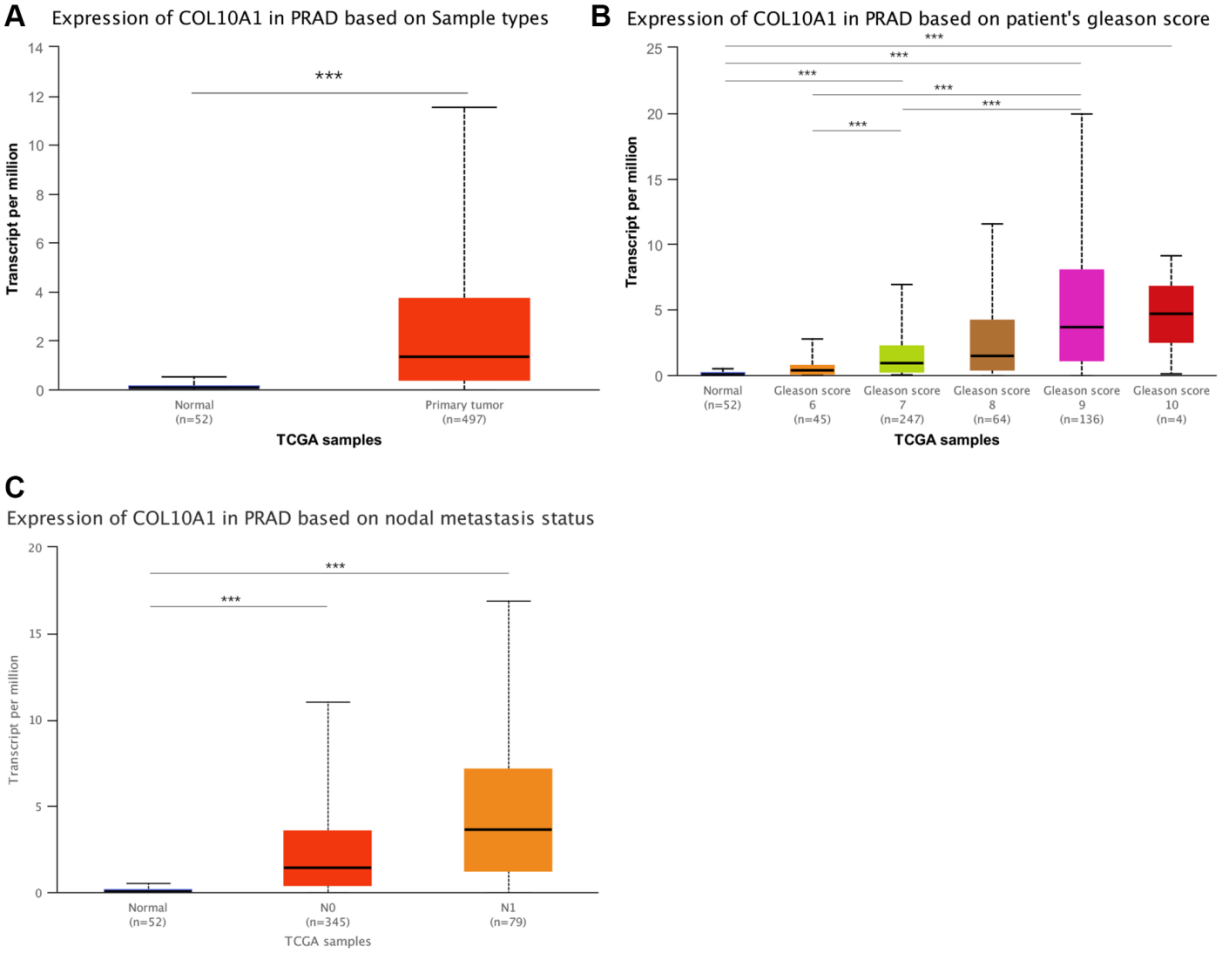
**An in-depth study of COL10A1 overexpression and clinical features of PRAD.** Results of the pairwise difference analysis of COL10A1 in PRAD (**A**). The relationship between COL10A1 overexpression in PRAD and Gleason scores and nodal metastasis (**B**, **C**).

In the above analysis, it was found that COL10A1 expression associated with the clinical features and prognosis of a variety of cancer types, and our team also found relationships between COL10A1 expression and immune cell infiltration, TMB and MSI. These were all highly associated with PRAD. Subsequently, the clinical features of high COL10A1 expression and PRAD were then further investigated using the UALCAN database. Figure 12A showed the results of COL10A1 expression in PRAD. Significantly, the aberrant expression of COL10A1 in PRAD was closely associated with the Gleason score. Higher COL10A1 expression corresponded to higher Gleason scores (Figure 12B). In addition, overexpression of COL10A1 in PRAD was closely associated with nodal metastasis (Figure 12C). All these studies indicated that COL10A1 is a key player in the pathogenesis of PRAD.

### COL10A1 is highly expressed in cell lines and tissues of prostate cancer

**Figure 13:**
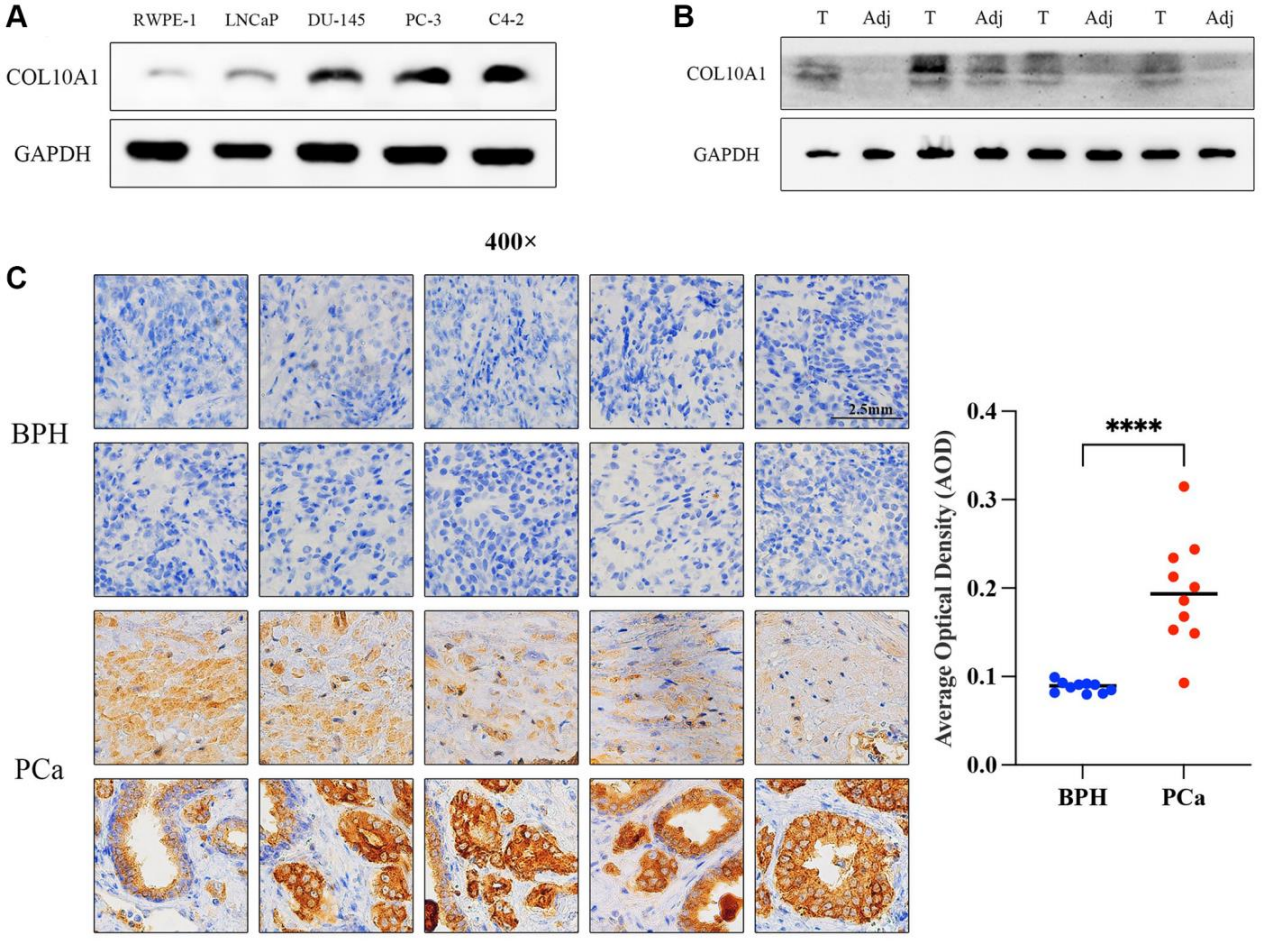
**COL10A1 highly expressed in prostate cancer cell lines and tissues.** The Western Blot assay was performed the expression of COL10A1 with RWPE-1, LNCaP, DU-145, PC-3 and C4-2 cell lines (**A**). The Western Blot assay was performed the expression of COL10A1 with 4 pairs of prostate cancer specimens and adjacent tissues specimens (**B**). Immunohistochemistry analysis of COL10A1 protein expression in 10 pairs of the prostate cancer tissues and BPH tissues (**C**).

To discover the expression level of COL10A1 in normal prostate cells and prostate cancer cells, the expression level of COL10A1 was preliminarily discussed in GEO database GSE60329 (Supplementary Figure 7). Western Blot was performed in the RWPE-1, LNCaP, DU-145, PC-3 and C4-2 cell lines. It was revealed that at the protein level, except for LNCap, the expression of COL10A1 in the other three prostate cancer cell lines were markedly upregulated compared to RWPE-1 (normal prostate cells) (Figure 13A). Additionally, the expression level of COL10A1 in 4 pairs of prostate cancer tissues and adjacent tissues was observed via Western Blot. The expression level of COL10A1 was markedly higher in prostate cancer tissues compared to adjacent tissues (Figure 13B). We performed immunohistochemistry staining (IHC) for COL10A1 on 10 pairs of prostate cancer patient and benign prostate hyperplasia (BPH) tissues specimens. The outcomes indicated that COL10A1 was expressed at a much higher level in the prostate cancer samples than it was in the BPH tissues (Figure 13C). In summary, the findings demonstrated the overexpression of COL10A1 in prostate cancer, corroborating the observations from the bioinformatic analysis and providing a foundation for subsequent experiments.

### COL10A1 siRNA suppresses prostate cancer cells growth, migration, and invasion

**Figure 14:**
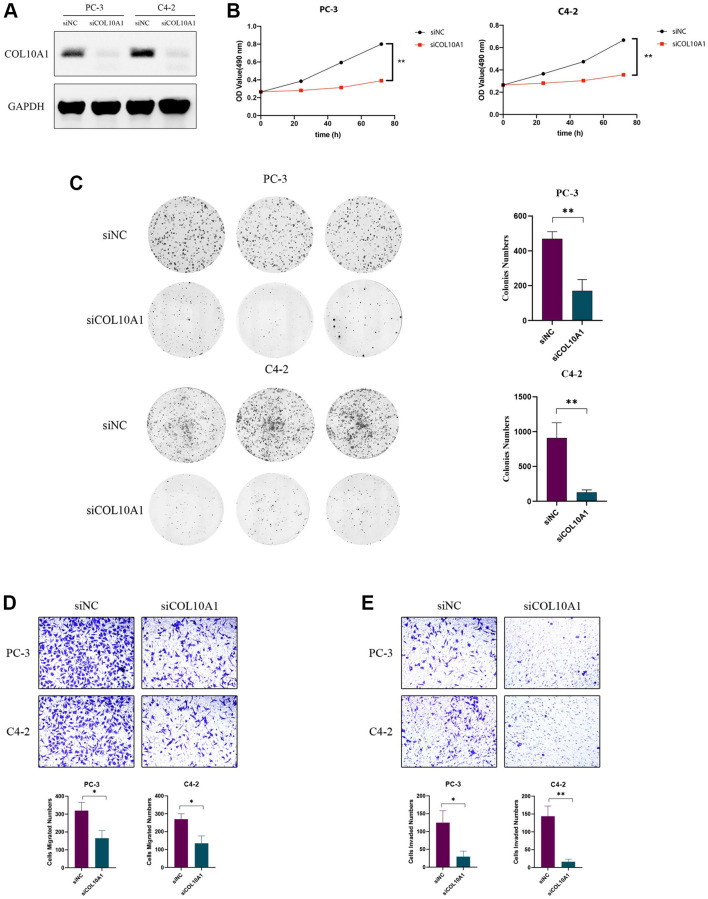
**Knockdown of COL10A1 inhibits proliferation, migration, and invasion of prostate cancer cells.** Western blotting analysis of the indicated proteins in PC-3 and C4-2 cells transfected with the indicated siRNA (**A**). CCK-8 assay was performed to detect the cell viability of PC-3 and C4-2 cells that knocked down for COL10A1 by siRNA (**B**). Colony formation assay was performed to detect the cell growth of PC-3 and C4-2 cells that knocked down for COL10A1 by siRNA (**C**). Transwell assays of PC-3 and C4-2 cells with knockdown of COL10A1 were showed the ability of migration and invasion (**D**, **E**).

To investigate the impact of COL10A1 on cancer cells, PC-3 and C4-2 cells were transduced with COL10A1 siRNA. The Western Blot assay result indicated that the protein level of COL10A1 was significantly downregulated in cells transduced with si-COL10A1 in comparison to those transduced with siNC (Figure 14A). The CCK-8 and colony formation experiments demonstrated that the targeted knockdown of COL10A1 inhibited the proliferation of PC3 and C4-2 cells to a greater extent than the cells transduced with siNC (Figure 14B, 14C). Likewise, knockdown of COL10A1 markedly blocked the migration of PC-3 and C4-2 cells in the transwell migration experiment (Figure 14D). Moreover, downregulation of COL10A1 remarkably reduced the invasive ability of PC-3 and C4-2 cells as in comparison to those transduced by siNC (Figure 14E). Together, these findings suggested that attenuating the COL10A1 can greatly suppress the aggressiveness of prostate cancer cells.

## DISCUSSION

COL10A1 was found to be enriched in the tumor as a part of the collagen family [1]. The ectopic expression of members of the collagen family can change the characteristics of the epithelial cells and re-model the morphology and function of the epithelial cells. This process promotes the detachment of cancer cells from the original tumor mass and the cell invasiveness needed for metastasis to occur, allowing cancer cells to migrate into the bloodstream or lymphatic system and colonise distant sites [14, 15].

COL10A1 can also slow down the autophagy pathways [16, 17]. Previous work has demonstrated that COL10A1 expression was upregulated in a variety of tumor tissues, such as lung cancer [18], gastric cancer [19], and breast cancer [20]. Same as before, our latest study showed that COL10A1 was strongly upregulated in the majority of cancers, and that high COL10A1 expression was associated with a worse cancer prognosis.

In this study, we jointly analyzed the expression level of COL10A1 in pan-cancer using R software as well as online databases. Our team demonstrated that COL10A1 expression was strongly different between most cancers and normal tissues. Its expression was markedly upregulated in BLCA, BRCA, CHOL, COAD, ESCA, HNSC, LUAD, LUSC, PCPG, PRAD, READ, STAD, THCA and UCEC in comparison to normal tissues. Our findings were further supported by the TIMER database.

Also, a pan-cancer-based survival analysis demonstrated that COL10A1 was a risk factor for most cancers, and an elevated COL10A1 expression usually indicated a poor prognosis for patients (especially in KIRC, KIRP, STAD, PAAD, SARC, PRAD and UCEC). Furthermore, increased expression of COL10A1 was usually correlated with late stage of cancers, which was consistent with the results of survival analysis. In particular, increased expression of COL10A1 in PRAD was also associated with higher Gleason score and nodal metastasis. The above findings may suggest that COL10A1 could be applied as a potential biomarker, especially in PRAD.

In recent years, genomic mutations have gained popularity among researchers. Researchers have found that genomic mutations are the key players in the development and progression of cancer, as well as in chemotherapy resistance [21]. For example, recent studies have shown that genetic mutations lead to an increase in Rac1 activity or expression, which promotes cancer cell development, progression, and leads to a poor patient prognosis [22]. In our present work, we found that mutations in COL10A1 were most commonly found in endometrial cancer (>10%).

The TME includes tumor cells, some immune cells surrounding the tumor cells, and stromal cells, which is the microenvironment for tumor cell development and survival [23]. Among the components that make up the TME, immune cells, which are responsible for supporting the tumor, play a key role in this tumor environment [24]. Although under normal circumstances immune cells can identify and destroy neoplastic cancer cells during cancer immunosurveillance, they may be affected by different factors present in the tumor during cancer immune editing. The fact that immune cells can act as guardians (antitumor immunity) or as bystanders or supporters of the tumor (pro-tumor immunity) makes them a “double-edged sword” in TME. The infiltrating immune cells, such as B cells, CD4+ T cells, CD8+ T cells, macrophages, and neutrophils, secreted a variety of factors that affected TME and regulated tumor behavior and anti-cancer capacity. Many studies have discovered that immune infiltration of tumors is associated with the prognosis of cancer [25]. Interestingly, our research showed that all immune cells were positively associated with increased COL10A1 expression in PRAD, which predicted a potential immunological role of COL10A1 in PRAD.

Using the TISIDB database, we continued to investigate the association between COL10A1 level and immunophenotyping and molecular typing of different types of cancer. The findings showed that there were marked differences in the expression of COL10A1 in different immunological and molecular subtypes of several cancers, which can be used as a marker to distinguish the immune and molecular subtypes of tumors, thus guiding clinicians for precise treatment. Furthermore, our team found that COL10A1 was strongly correlated with two immunotherapy biomarkers, TMB and MSI, in a variety of cancers. TMB not only provided a useful estimate of Tumor mutational burden, but also effectively predicted the efficacy of immunotherapy in many cancers [26, 27]. MSI was a promising indicator for immunotherapy, defined as a highly mutant phenotype as a result of defects in DNA mismatch repair [28].

In this study, COL10A1 was identified as being correlated with TMB and MSI in cancers. In OV, PRAD, THYM and UCEC, the expression of COL10A1 was positively associated with TMB, indicating that the higher expression of COL10A1 was associated with a higher mutation in the above tumors. In COAD, SARC and UCEC, the expression of COL10A1 was positively associated with MSI. In contrast, in BRCA, HNSC, KIRC, KIRP and LUSC, COL10A1 expression level was significantly negatively associated with both TMB and MSI.

Thus, COL10A1 may affect tumourigenesis by participating in the process of genetic alteration. The findings also suggested that COL10A1 may be a significant biomarker for the treatment and prognosis of several cancers. Furthermore, studies of immunotherapy cohorts have shown that patients with a lower level of COL10A1 expression are more likely to benefit from immunotherapy in urothelial cancer cohorts. Consequently, COL10A1 is of great value as a biomarker for immunotherapy of carcinoma. Furthermore, our team observed an extremely strong relationship between abnormally high COL10A1 expression and clinical characteristics of PRAD patients as well as patient prognosis, especially in close association with Gleason score and nodal metastasis. Our experiments demonstrated that the knockdown of COL10A1 leads to frustration of biological behaviors such as proliferation, migration and invasion of prostate cancer cells. All of this suggests that COL10A1 may have potential clinical value for prognostic evaluation and follow-up treatment of PRAD. Currently, the exact mechanism by which high expression of COL10A1 promotes the progression of PRAD is unclear. These findings suggest a prospective role of high COL10A1 expression in the development and progression of PRAD.

The present research explored the impact of aberrant COL10A1 expression on cancer progression, as well as on patient prognosis, and warrants further research from investigators. There were limitations in our research. Firstly, we did the bioinformatics analysis based on public databases using the R software. In addition, our results and conclusions have not been validated experimentally or prospectively in the clinical setting. Therefore, COL10A1 needs to be further investigated for its biological functions *in vivo* and *in vitro*.

## MATERIALS AND METHODS

### Data collection

Gene expression profiles and corresponding clinicopathologic feature information for pan-cancer were acquired from the TCGA database [29]. Besides, the data of somaclonal variation were acquired from TCGA database. To further understand the genetic alterations of COL10A1 in human cancers, we retrieved mutations and copy number variants of COL10A1 for visibility using the cBioPortal database (https://www.cbioportal.org) [30]. In addition, the PRAD GEO datasets GSE60329 was used to validate COL10A1 expression (GEO) (https://www.ncbi.nlm.nih.gov/geo/) (https://www.ncbi.nlm.nih.gov/geo/) databases [31]. The whole RNA-seq data were normalised by log2 transformation.

### Analysis of the expression of COL10A1 in pan-cancer

The analysis of gene differential expression was analyzed by using the limma package with FPKM datatype in R software (R-4.1.3) to identify if COL10A1 expression differs between carcinoma and the corresponding normal adjacent tissues. Similarly, we also examined the relationship of COL10A1 expression with other clinical data (age, gender, and tumor stage). Our team also used The Tumor Immune Estimation Resource (TIMER, https://cistrome.shinyapps.io/timer/) database to investigate the expression profiles of COL10A1 in pan-cancer tissues (ns, *p* ≥ 0.05; ^*^p < 0.05; ^**^p < 0.01; ^***^p < 0.001) [32]. The TIMER database is normalized using log2 TPM. The Sangerbox website (http://sangerbox.com) was utilized to perform the expression of COL10A1 in pan-cancer after combining the TCGA and GETx datasets. Our team downloaded the harmonised and standardised pan-cancer dataset: TCGA TARGET GTEx (PANCAN, *N* = 19131, G = 60499) from the UCSC (https://xena.ucsc.edu/) database, from which we further extracted the expression data of ENSG00000123500 (COL10A1) gene in various samples. Our team further extracted the expression data of ENSG00000123500 (COL10A1) gene in each sample, and we screened the samples from: Solid Tissue Normal, Primary Solid Tumor, Primary Tumor, Normal Tissue, Primary Blood Derived Cancer - Bone Marrow, Primary Blood Derived Cancer - Peripheral Blood. Furthermore, our team performed log2(TPM+0.001) transformation for each expression value. Finally, we also excluded cancers with less than 3 samples in a single cancer, and obtained expression data for 34 cancers.

### Analysis of the clinical correlation and prognosis of COL10A1 in various cancers

Using Cox regression analysis, the correlation between COL10A1 expression and survival outcomes including overall survival (OS), disease-specific survival (DSS), disease-free survival (DFS) and progression-free survival (PFS) was assessed in patients using the TCGA database. When the hazard ratio exceeded 1 (HR > 1), it suggested that the factor of the exposure (COL10A1 expression) was a promoter of the positive event (death). K-M analysis was performed by the R packages “survival” and “survminer”. The R packages “survival” and “forestplot”, together with the Sangerbox website, were used to implement forestplot in Cox regression. Furthermore, we further validated the association between COL10A1 expression and survival in the Kaplan-Meier plotter (https://kmplot.com/analysis/) and PrognoScan (http://dna00.bio.kyutech.ac.jp/PrognoScan/index.html) regarding clinical results such as OS, DSS, DFS and PFS [33, 34]. Finally, we applied the UALCAN (http://ualcan.path.uab.edu) database to analyze the expression and clinical relevance of COL10A1 in Prostate adenocarcinoma [35, 36]. *P*-values of less than 0.05 were considered to be significant. The data format for this section is FPKM.

### Analysis of the expression of COL10A1 in relation to clinical features by using TISIDB

TISIDB (http://cis.hku.hk/TISIDB/index.php) is a web-based tumor immunoassay resource that incorporates a large number of data sets from the TCGA database [37]. It contains extensive data on cancer immunity. TISIDB was used to investigate the relationship between COL10A1 levels and molecular and immunological subtypes. The violin plot illustrates the above. The Spearman’s correlation analysis test was used to examine the relationship between COL10A1 and the clinicopathological features.

### Correlation analysis of COL10A1 expression in immune-related factors

The TME contains cancer cells, immune cells and stromal cells, which make up the microenvironment in which the cancer cells develop and survive [38]. The immune and stromal cell scores were derived using the ESTIMATE package, which can be used to estimate the extent of stromal and immune cell infiltration and tumor purity in pan-cancerous tissue [39, 40]. Specifically, the levels of stromal cells and immune cells in the tumor microenvironment influenced the biological behaviour of the cancer cells [41]. Subsequently, our team utilized the “Gene” module of the TIMER database to explore the association between the level of COL10A1 expression in the TCGA database and the level of immune infiltration in various cancer types. In addition, earlier studies have also shown that TMB and MSI are strongly linked to the immune response of tumor cells. Therefore, our team used radar plots to show the relationship between the level of COL10A1 expression and TMB and MSI.

### Immunotherapy analysis

Immunotherapeutic analysis was used by investigators to more clearly understand the mode of action between gene expression patterns and immunotherapeutic effects.

Overall, immunotherapy can produce four results: complete remission, partial remission, disease progression, and disease stabilization. In our study, people who showed complete or partial remission were classified as responders and compared with non-responders who demonstrated evidence of disease progression or stabilization. Finally, three datasets, GSE78220, GSE67501 and IMvigor210, were selected to compare the variation in COL10A1 gene levels between patients in the responder and non-responder groups after immunotherapy. The data for all three sets of data were obtained from the Gene Expression Omnibus (GEO) database [42–44]. The R package “limma”, “ggplot2” and “ggpubr” were used to plot box plots for the three treatment groups and to give *p*-values for comparisons between groups.

### Specimen collection and cell culture

Prostate cancer and matched paracancerous samples were collected from operated specimens of prostate cancer patients from the Department of Urology, Second Hospital of Tianjin Medical University, and stored in liquid nitrogen as soon as possible after excision. Our research had the approval of the ethics committee of the Second Hospital of Tianjin Medical University. The human ethical approval number is KY2023K144. Each patient was the subject of an informed consent form. The human prostate cancer cell lines PC-3, DU-145, LNCaP, C4-2 and the RWPE-1 normal prostate cell line were obtained from the American Type Culture Collection. All of these cells have been frozen, revived and grown in culture by the Tianjin Institute of Urology. All cell lines were incubated in DMEM or RPMI 1640 medium (Gibco BRL Life Technologies, Waltham, MA, USA) which contained 10% FBS (Gibco BRL Life Technologies) and 1% antibiotics (Gibco BRL Life Technologies). The cells were all cultured in a humidified incubator at 37°C with 5% CO_2_.

### Cellular transfection

Small interfering RNAs were purchased from Shanghai Gene Pharma company. Cells were transfected with the appropriate small interfering RNA using the Lipofectamine 3000 Kit (Thermo Fisher Scientific, USA) following the instructions of the manufacturer. The medium was renewed after 8 hours of incubation. The siRNA sequences were shown below: siCOL10A1, 5′-GCAUGUGAAAGGGACUCAUTT-3′ sense and 5′-AUGAGUCCCUUUCACAUGCTT-3′ antisense; negative control (NC), 5′-UUCUCCGAACGUGUCACGUTT-3′ sense and 5′-ACGUGACACGUUCGGAGAATT-3′ antisense.

### Western blot

Proteins were isolated from cells or tissues using a protein extraction kit (CWBIO, Beijing, China). Protease inhibitors were added to the kit. The bicinchoninic acid test was applied to measure the protein concentration. The proteins were then isolated by 10% sodium dodecyl sulphate gel electrophoresis (SDS-PAGE) and transferred to PVDF membranes. After being washed with TBS-T and being blocked with 5% skimmed milk, the PVDF membranes were then incubated with a polyclonal antibody to COL10A1 (1:1000, Novus, USA). GAPDH (1:6000, Proteintech, Wuhan, China) was utilized as an internal loading control. To bind the corresponding primary antibody, the appropriate horseradish peroxidase (HRP)-conjugated secondary antibody was used. And then enhanced chemiluminescence (ECL) detection reagent was applied to identify protein bands. (Proteintech, Wuhan, China). Signals were detected using Tanon (Shanghai, China) chemiluminescence gel imaging system.

### Immunohistochemistry

Paraffin-embedded tissue sections (4 μm) were then placed on glass slides. Subsequently, the tissue sections were dewaxed and rehydrated. First, it was treated with 3% H_2_O_2_ for 10 minutes. Sections were then placed in sodium citrate (pH 6.0) for 15 minutes in a microwave oven. Cells were incubated with anti-COL10A1 (1:100, Novus, USA) at 4°C overnight after blocking with 5% bovine serum albumin (BSA) for 60 minutes. Staining results were visualized by DAB. Finally, photos were taken under a microscope (Olympus, Tokyo, Japan). We calculated the average optical density (AOD) values of COL10A1 in the tissue using the image-J IHC profiler and performed statistical analysis using GraphPad Prism9.

### CCK-8 assay

The CCK-8 kit (Apexbio, USA) was applied to assess the proliferation of prostate cancer cells. Logarithmic cells were collected and inoculated into 96-well plates. The cells were incubated for a period of 0, 24, 48 and 72 hours. After that, CCK-8 was then poured into the flask, and the incubation was allowed for another 4 hours. The enzyme-linked immunoassay analyser was used to read the absorbance of each plate at 490 nm.

### Clone formation assay

After COL10A1 was knocked down, PC-3 and C4-2 cells were seeded onto a 6-well plates at 500-1,000 cells/well. The cells were then cultured for 2 weeks. When there were more than 50 cells per colony, The medium was taken out and the PBS was washed two times. Tumor cells were stained with 0.1% crystal violet after 15 minutes treatment with 5 ml 4% paraformaldehyde. After washing three times with PBS, the colonies were counted.

### Trans-well assays

For the measurement of cell migration and invasion, transwell chambers were filters with a pore size of 8 μm. The transfected cancer cells were washed with PBS and resuspended in serum-free medium. Cells were seeded into trans-well chambers with (invasion assay) or without (migration assay) Matrigel matrix (BD, USA) following the manufacturer’s instructions, and then incubated with medium and 10% FBS in the bottom compartment and transferred to constant temperature incubators. After 48 hours of culturing, cells in the top compartment were swabbed, fixed in formaldehyde, stained with 0.5% crystal violet and photographed for cells that had migrated to the bottom surface.

### Statistical analysis

Expression of the COL10A1 gene was evaluated across various tumors using the Wilcoxon rank sum test. The association between COL10A1 expression and patient survival was examined through Kaplan-Meier analysis and univariate Cox proportional hazards regression analysis. To assess the link between COL10A1 expression and factors such as immune cell infiltration, tumor mutation burden (TMB), and microsatellite instability (MSI), Spearman’s correlation test was employed. The *t*-test was used to determine the significance of the differences between the experimental and control groups. A significance threshold of *P* < 0.05 was set for this analysis.

## Supplementary Materials

Supplementary Figures

Supplementary Tables
