# Supplementary material for: Experimental validation and pan-cancer analysis identified COL10A1 as a novel oncogene and potential therapeutic target in prostate cancer
Source: Aging (Albany NY). 2023 Dec 21;15(24):15134–60. doi: 10.18632/aging.205337 (PMC10781495; doi:10.18632/aging.205337)
Supplement: Supplementary Figures [file aging-15-205337-s001.pdf]

SUPPLEMENTARY FIGURES

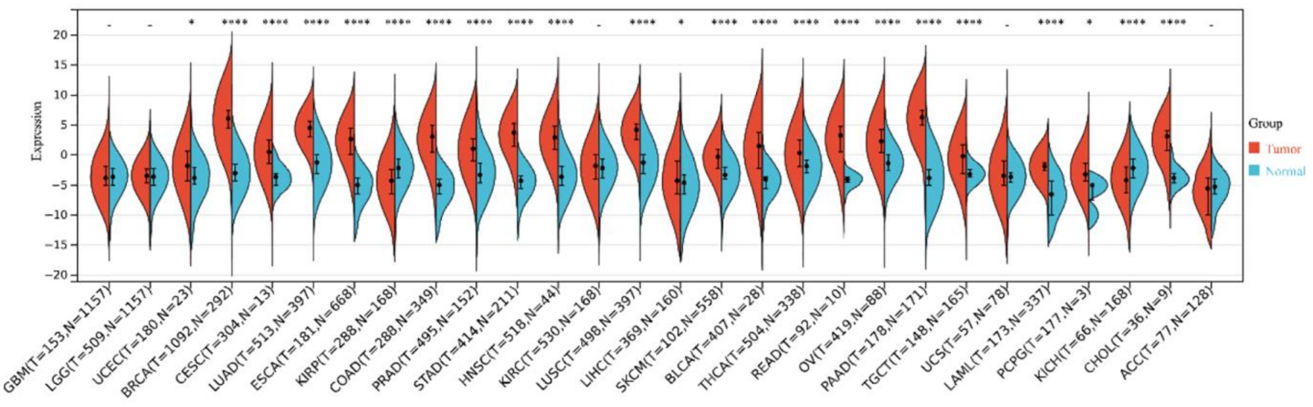

Supplementary Figure 1. The Sangerbox website analyzed the results of differential expression of COL10A1 in pan-cancer after combining data from TCGA and GTEx databases.

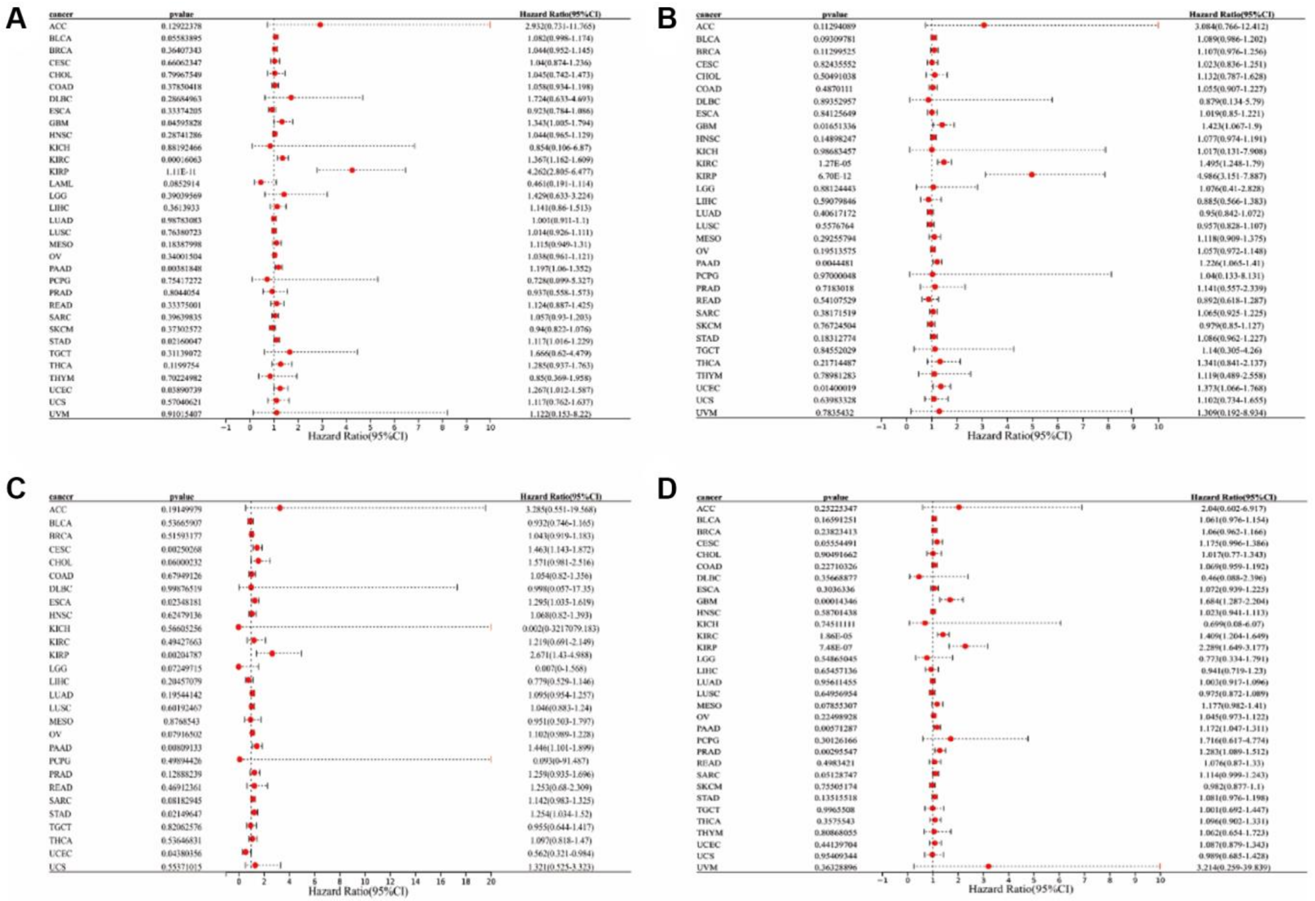

Supplementary Figure 2. Cox regression analysis of COL10A1 in pan-cancer. OS (**A**), DSS (**B**), DFS (**C**), PFS (**D**).

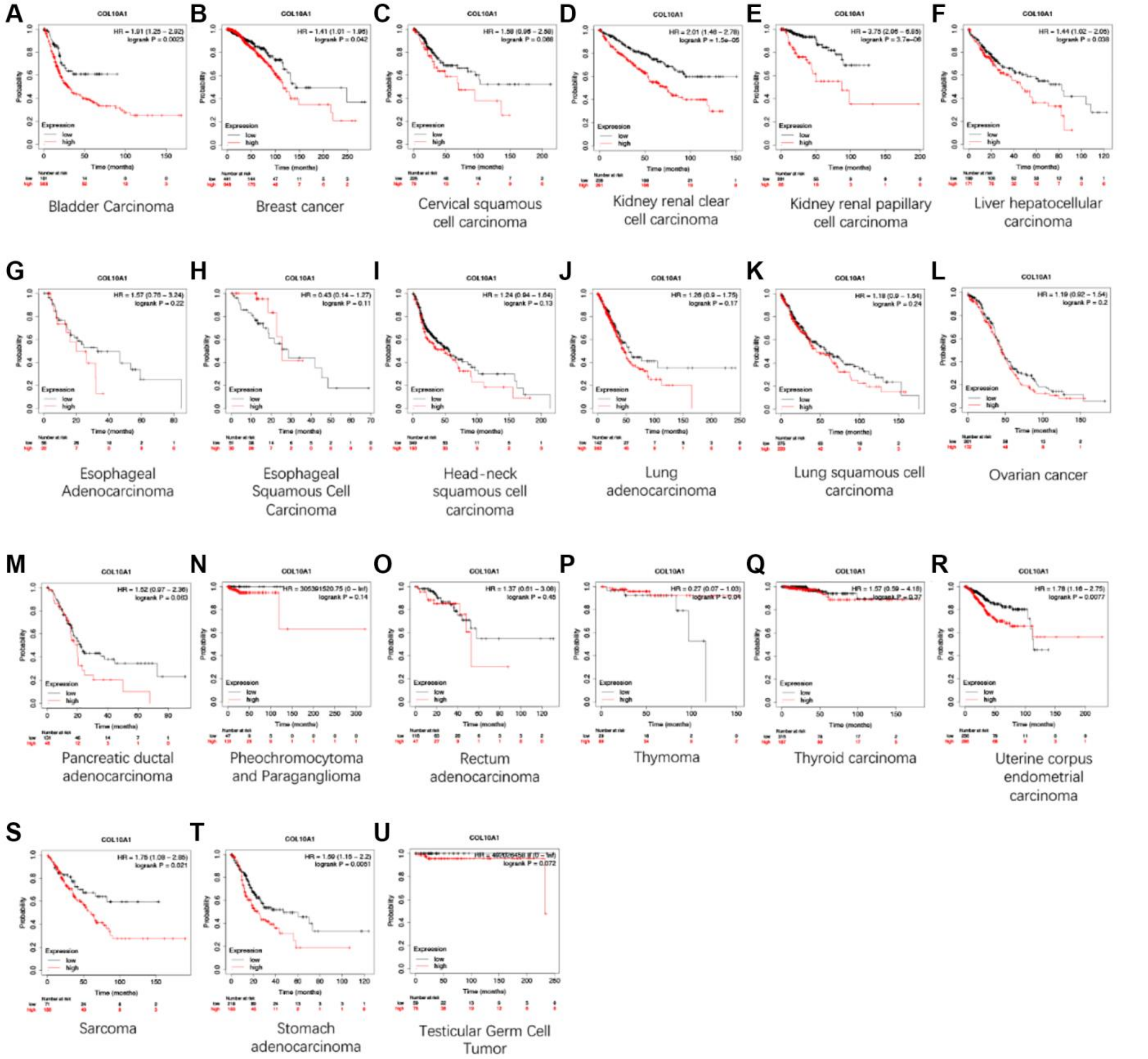

Supplementary Figure 3. The prognostic value of COL10A1 expression on overall survival time in pan-cancer was analyzed using Kaplan-Meier plotter (**A**–**U**).

A

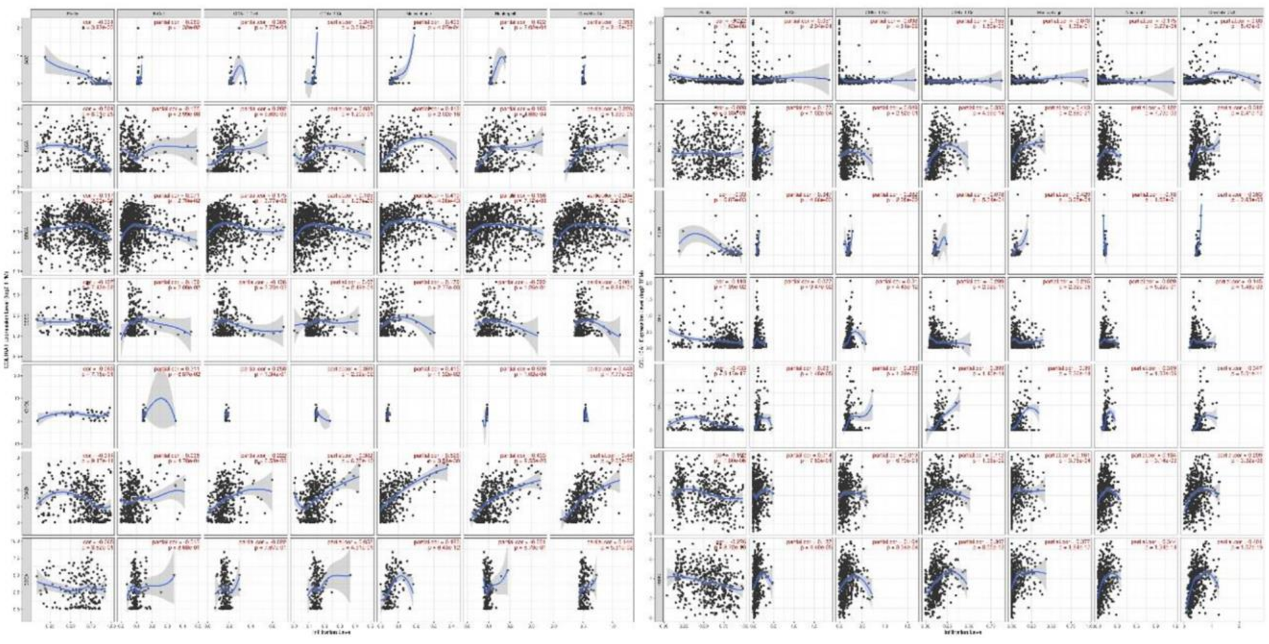

B

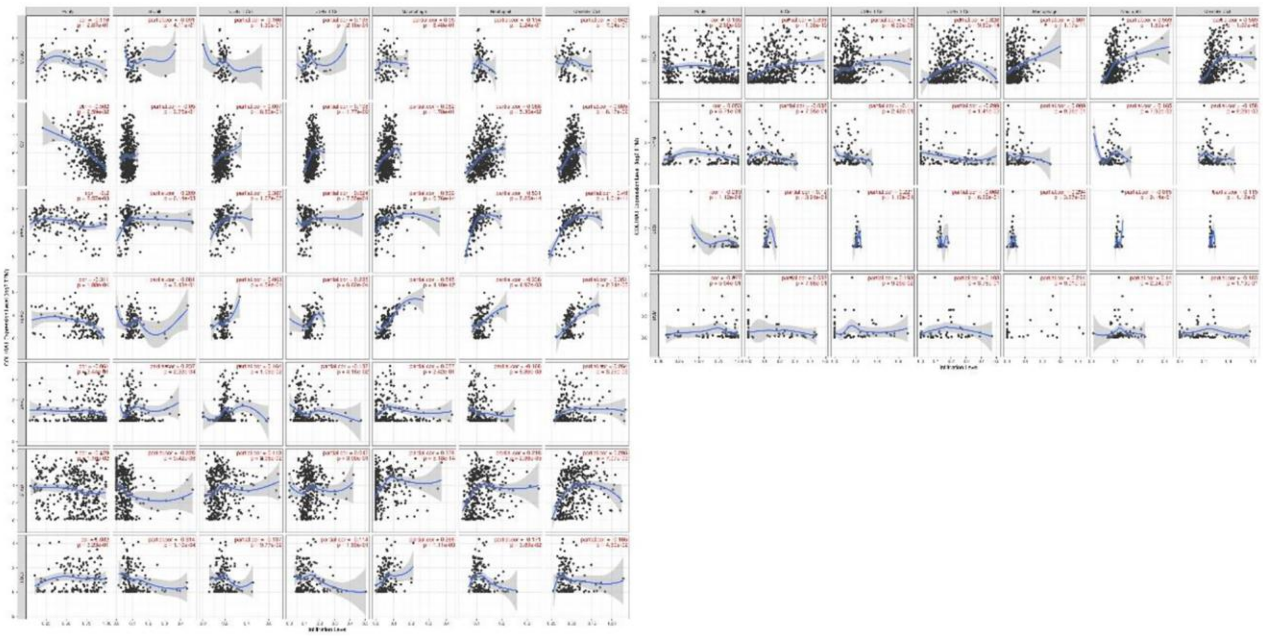

Supplementary Figure 4. (**A**, **B**) Results of the remaining immune cell infiltration.

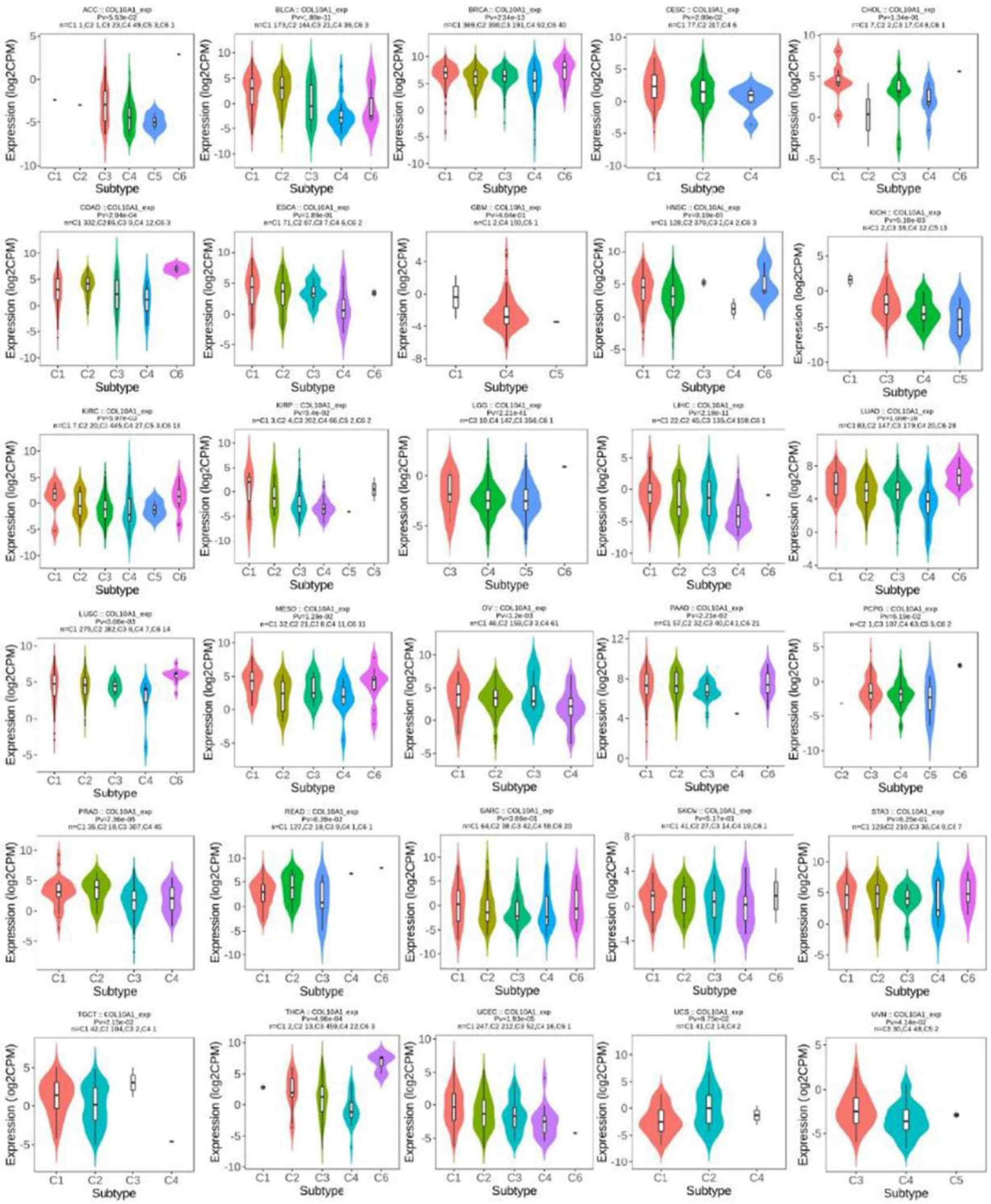

Supplementary Figure 5. TISIDB database on the relationship between pan-cancer COL10A1 expression and immunological subtypes.

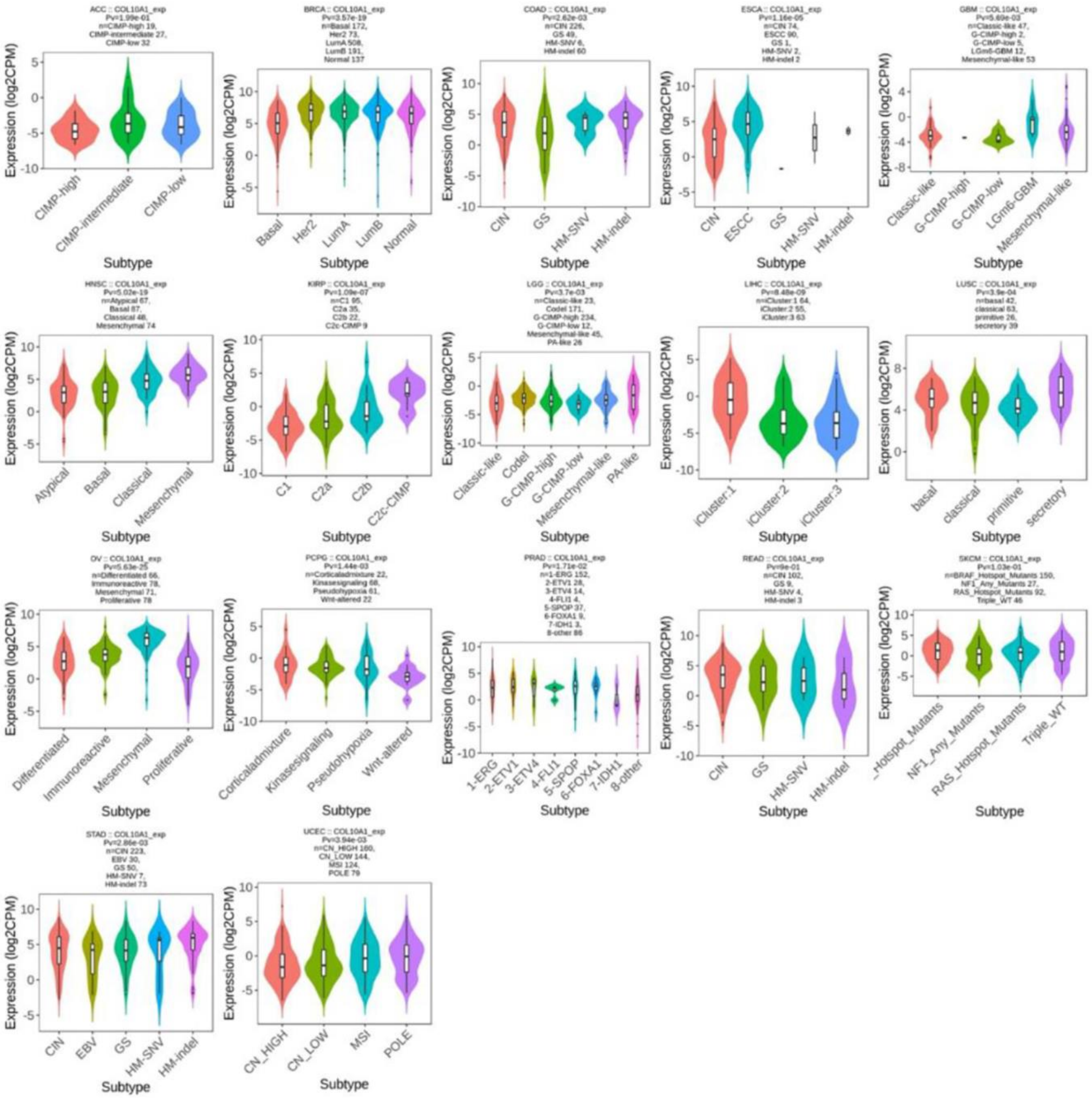

Supplementary Figure 6. TISIDB database on the relationship between pan-cancer COL10A1 expression and molecular subtypes.

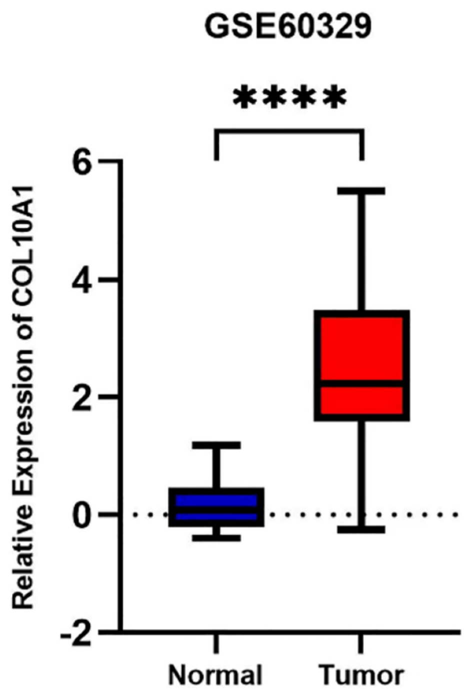

Supplementary Figure 7. Expression trends of COL10A1 in GEO databases GSE60329.
