# Supplementary material for: Experimental validation and pan-cancer analysis identified COL10A1 as a novel oncogene and potential therapeutic target in prostate cancer
Source: Aging (Albany NY). 2023 Dec 21;15(24):15134–60. doi: 10.18632/aging.205337 (PMC10781495; doi:10.18632/aging.205337)
Supplement: Supplementary Tables [file aging-15-205337-s002.pdf]

SUPPLEMENTARY TABLES

Supplementary Table 1. Abbreviations and details of the 33 cancer types used in this study.

Abbreviation	Detail
ACC	Adrenocortical carcinoma
BLCA	Bladder urothelial carcinoma
BRCA	Breast invasive carcinoma
CESC	Cervical squamous cell carcinoma and endocervical adenocarcinoma
CHOL	Cholangiocarcinoma
COAD	Colon adenocarcinoma
DLBC	Lymphoid neoplasm diffuse large B-cell lymphoma
ESCA	Esophageal carcinoma
GBM	Glioblastoma multiforme
HNSC	Head and neck squamous cell carcinoma
KICH	Kidney chromophobe
KIRC	Kidney renal clear cell carcinoma
KIRP	Kidney renal papillary cell carcinoma
LAML	Acute myeloid leukemia
LGG	Brain lower grade glioma
LIHC	Liver hepatocellular carcinoma
LUAD	Lung adenocarcinoma
LUSC	Lung squamous cell carcinoma
MESO	Mesothelioma
OV	Ovarian serous cystadenocarcinoma
PAAD	Pancreatic adenocarcinoma
PCPG	Pheochromocytoma and paraganglioma
PRAD	Prostate adenocarcinoma
READ	Rectum adenocarcinoma
SARC	Sarcoma
SKCM	Skin cutaneous melanoma
STAD	Stomach adenocarcinoma
TGCT	Testicular germ cell tumors
THCA	Thyroid carcinoma
THYM	Thymoma
UCEC	Uterine corpus endometrial carcinoma
UCS	Uterine carcinosarcoma
UVM	Uveal melanoma

Supplementary Table 2. The relationship between COL10A1 gene expression and the prognosis of different cancers in PrognoScan.

Gene	Dataset	Cancer type	Endpoint	*N*	Cox *P*-value	HR	95% CI (low-high)
COL10A1	GSE2034	Breast cancer	DFS	286	0.00989	1.3	1.06–1.58
COL10A1	GSE17536	Colorectal cancer	DFS	145	0.000163	1.54	1.23–1.93
COL10A1	GSE14333	Colorectal cancer	DFS	226	0.000307	1.39	1.16–1.66
COL10A1	GSE17537	Colorectal cancer	DFS	55	0.037971	1.27	1.01–1.60
COL10A1	GSE26712	Ovarian cancer	DFS	185	0.038843	1.22	1.01–1.48
COL10A1	GSE30929	Soft tissue cancer	DFS	140	0.04401	1.29	1.01–1.65
COL10A1	GSE13507	Bladder cancer	DSS	165	0.002572	1.83	1.24–2.71
COL10A1	GSE3494-GPL96	Breast cancer	DSS	236	0.039744	0.77	0.60–0.99
COL10A1	GSE17536	Colorectal cancer	DSS	177	0.000248	1.37	1.16–1.63
COL10A1	GSE13507	Bladder cancer	OS	165	0.035124	1.51	1.03–2.22
COL10A1	GSE4412-GPL96	Brain cancer	OS	74	0.034852	1.45	1.03–2.06
COL10A1	GSE17536	Colorectal cancer	OS	177	0.003222	1.23	1.07–1.41
COL10A1	GSE31210	Lung cancer	OS	204	0.030322	1.69	1.05–2.71
COL10A1	GSE9891	Ovarian cancer	OS	278	0.001333	1.17	1.06–1.28
COL10A1	GSE1379	Breast cancer	RFS	60	0.039888	1.76	1.03–3.01
COL10A1	GSE31210	Lung cancer	RFS	204	0.0119	1.55	1.10–2.17
